# Aluminosilicates at different levels in rye litter and feed affect the growth and meat quality of broiler chickens

**DOI:** 10.1007/s11259-021-09827-x

**Published:** 2021-09-03

**Authors:** Mirosław Banaszak, Jakub Biesek, Marek Adamski

**Affiliations:** Department of Animal Breeding and Nutrition, Faculty of Animal Breeding and Biology, Bydgoszcz University of Science and Technology, Mazowiecka 28, Bydgoszcz, 85-084 Poland

**Keywords:** Broiler, Rye litter, Zeolite, Halloysite, Growth, Meat quality

## Abstract

Litter sanitation treatments and feed supplements that stimulate bird growth. The aim of this study was to analyse the effects of zeolite (z) and halloysite (h) in feed and rye straw litter on growth performance, meat quality in chickens. 500 males Ross 308 were allocated to 5 groups (10 replicates). Feed for groups 2–5 was supplemented with halloysite and zeolite (25:75). The content of aluminosilicates in the feed was different depending on the feeding phase: 0.5, 1, 1.5, or 2%. The following doses were used in litter: 2, 0.800 kg/m^2^ h; 3, 0.400 kg/m^2^ h and 0.400 kg/m^2^ z; 4, 0.800 kg/m^2^ z; 5, 0.200 kg/m^2^ h, and 0.600 kg/m^2^ z. Growth, and meat quality were analysed. Body weight and body weight gain were higher in groups 2–5 than in group 1, while feed intake was lower in groups 1 and 2 (*p* < 0.05). Body weight, the weight of carcass, and most of its elements were higher in the experimental groups (*p* < 0.05). Breast muscles from group 1 were characterised by better water-holding capacity and higher protein content, while those from 4 had higher content of collagen and water (*p* < 0.05). Breast muscles from group 3 were characterized by lower yellowness than in 2 (*p* < 0.05). Leg muscles from group 1 were characterized by lower redness than in 4 (*p* < 0.05). Water-holding capacity was better in group 3 and protein content was higher in 2 (*p* < 0.05). The content of fat in leg muscle was lower in all experimental groups compared to control (*p* < 0.05). This indicates the suitability of aluminosilicates in poultry management practice, with better growth and meat quality.

## Introduction

Dynamically growing poultry industry is facing new challenges associated with the health of birds, and effective production and delivery of good quality safe meat, free from antibiotic residues (Hafez and Attia 2020). The issue of antibiotics use in animal production concerns many countries where restrictions are introduced, but antibiotics can be replaced with alternative agents improving production performance and meat quality, as well as the biosecurity on poultry farms (Selaledi et al. 2020). The safety of production is also related to animal welfare, which allows for the rearing of a healthy flock, and thus production of good quality meat (Iannetti et al. 2020). The antibiotic-free production of broiler chickens is challenging for many reasons, including consumer expectations, bird quality and environmental impact, and the most popular alternative agents are probiotics, prebiotics, and enzymes (Haque et al. 2020). As concluded by other authors (Haque et al. 2020; Roth et al. 2019), alternatives should ensure an appropriate volume of production, ensure consumer health and environmental protection, and therefore the search is focused on natural agents with beneficial effects on the animal body. An interesting solution may be aluminosilicates (natural zoelites), which are used in two areas: as agents ensuring good sanitary conditions, and as a feed additive to improve growth performance and meat quality (Andronikashvili et al. 2014). Aluminosilicates are natural minerals adsorbing ammonia and mycotoxins, which improves the quality and biosecurity of poultry production (Huff et al. 1992; Wlazło et al. 2016). Schneider et al. (2017) reported that the addition of zeolites in the diet of laying hens improved egg quality and had a positive effect on the growth performance of broiler chickens, as well as on the quality of their litter and even intestinal health. Natural zeolites added to feed and litter (5 g/kg of feed; 100 g/kg of litter) when used alone had no effect on growth performance or dressing percentage (Schneider et al. 2006). Halloysite is less popular, but research was conducted on the use of this mineral as an agent reducing odour emissions in poultry production, and the results revealed its beneficial effects (Korczyński et al. 2013). According to Shariatmadari (2008), findings on the positive effect of zeolites on the quality of production of broiler chickens are inconclusive, but there are some implications supporting this solution with respect to the sanitary conditions on poultry farms and the growth of chickens. These authors also suggested that doses of aluminosilicates should be adjusted with consideration of the production system, type of litter, and even the age and sex of birds. The available literature on the effects of aluminosilicates in feed and litter on meat quality in broiler chickens is limited, especially with respect to their combined use, so studies in this area seem to be fully justified.

## Material and methods

The aim of this study was to analyse and compare the effects of aluminosilicates in feed and chopped rye straw litter on growth performance, carcass traits, physicochemical parameters, and meat quality in broiler chickens reared on a large poultry farm.

The presented research is part of a project “Safe Farm – innovative products, processes and marketing in the production of broiler chickens”, implemented in 2020–2022 and co-financed from the European Agricultural Fund for Rural Development: Europe investing in rural areas” in cooperation with the Agency for Restructuring and Modernisation of Agriculture (Poland).

The study concerned the analysis of physicochemical characteristics of meat quality obtained from broiler chickens managed on a commercial farm. Growth performance was controlled by the farm owner in cooperation with the research team. Therefore, according to Directive no. 2010/63/EU, the study did not require approval from a Local Ethics Committee. No approval was required under Resolution no. 13/2016 of the National Ethics Committee for Animal Experiments of 17 June 2016. Slaughter of birds was done according to the Council Regulation (EC) No 1099/2009 of 24 September 2009 on the protection of animals at the time of the killing. All methods in the presented experiment were carried out in accordance with relevant guidelines and regulations.

### Bird management

The study was carried out on a large commercial broiler chicken farm. A separate experimental zone was established in a farm building, stocked with 500 Ross 308 broiler chickens assigned to 5 equal-size groups, in 10 replicates per group (10 birds per group). The separate experimental zone indicates that the studies were conducted according to standards in poultry experiments and the intended purpose was to indicate that the experiment was conducted on a large farm to demonstrate the nature of the R + D studies, in collaboration with broiler chicken producers. The study site had no effect on any disorders and rearing was fully controlled. The management of broiler chickens was compliant with relevant standards and recommendations (Aviagen 2015). Birds were kept on chopped rye straw litter for 42 days. In the study, litter material in the form of rye straw litter was used due to its universality and general availability from the point of view of poultry producers. The use of the addition of aluminosilicates is a response to the search for methods to raise standards in poultry production, which is currently related to the trend of using natural resources, taking into account the cost-effectiveness. Group 1 was the control, where standard management was applied without experimental factors. Groups 2–5 received different levels of aluminosilicates (halloysite and zeolite) in feed and litter. Chicken diet was based on commercially available feed and consisted of 4 feeding phases: starter, grower 1, grower 2 and finisher. Feed for groups 2–5 was supplemented with halloysite and zeolite in the proportion 25:75. The content of aluminosilicates in the feed was different depending on the feeding phase: 0.5,1, 1.5, or 2% (phases 1 to 4). Characteristics of groups and data on the addition of aluminosilicates in feed and litter are presented in Table 1. The basic chemical composition of feed for the control and experimental groups in each feeding phase is presented in Table 2. The feeds were purchased from commercial companies producing feeds for broiler chickens. The chemical composition declared for the applied mixtures fully corresponds to the Ross 308 chicken feeding standards, and the data presented in the Table 2 are analytical. The feeds were iso-protein and isocaloric. The aluminosilicates were added to the feed at the factory level of the feed company. The feed was in the form of granules. The test additive was mixed in the feed pellets. The chemical composition and physical properties of zeolite are described in the work of Biesek et al. (2021). In the presented work and in the cited source, aluminosilicates from the same supplier were used. Zeolite characterized with a specific surface area of 30–60 m^2^/g, a bulk density of 1.60–1.80 kg/m^3^, and weight of 2.20–2.44 kg/m^3^. Silicon dioxide (SiO_2,_ 65–71.30%) and aluminium oxide (Al_2_O_3_, 11.50–13.10%) were marked. Zeolite contains calcium oxide (CaO, 2.70–5.20%), potassium oxide (K_2_O, 2.20–3.40%), iron (III) oxide (Fe_2_O_3_, 0.70–1.90%), magnesium oxide (MgO, 0.60–1.20%), sodium oxide (Na_2_O, 0.20–1.30%), titanium oxide (TiO_2_, 0.10–0.30%), and 4.80–5.40% of Si/Al. Zeolite also contained minerals such as clinoptilolite (84%), cristobalite (8%), mica clay (4%), plagioclase (3–4%), rutile (0.10–0.30%), and traces of quartz. Halloysite composition contains 13.00% aluminium (Al), 12.00% silicon (Si), 0.40% calcium (Ca), 0.30% magnesium (Mg), 0.10% sodium (Na), 0.08% potassium (K), 0.30% phosphorus (P), 9.00% iron (Fe), 1.00% titanium (Ti), and 0.20% manganese (Mn). The specific surface area of halloysite is 65–85 m^2^/g and the bulk density is 0.70–0.85 g/cm^3^. Zeolite and halloysite added to the litter were in a powdery, dusty form.

**Table 1:** Description of broiler chickens used in the experiment

Group no	*n* birds	Replicates (pen x birds)	Addition in litter* (kg/m^2^)	Addition in feed
1	100	10 × 10	None	None
2	100	10 × 10	0.800 h^1^	halloysite and zeolite to feed in proportion 25:75; starter, 0.5%; grower 1, 1%; grower 2, 1.5%; finisher, 2%
3	100	10 × 10	0.400 h / 0.400 z^2^	
4	100	10 × 10	0.800 h	
5	100	10 × 10	0.200 h / 0.600 z	

^1,^ h, halloysite; ^2^, z, zeolite

*, weight per treatment; aluminosilicates were applied on 5 dates: days 1, 10, 20, 30, 35 of rearing

**Table 2:** Analytical composition of feeds for broiler chickens, 4 feeding phases

Constituent [%]	STARTER		GROWER 1		GROWER 2		FINISHER	
	C(1)^1^	E(2–5)^2^	C(1)	E(2–5)	C(1)	E(2–5)	C(1)	E(2–5)
Dry matter	88.70	87.74	88.85	89.25	89.24	88.58	88.61	88.71
Crude ash	7.74	7.52	5.51	5.12	5.31	5.57	5.13	5.86
Crude protein	20.75	21.17	19.96	21.92	19.37	20.03	18.30	18.76
Crude fat	5.08	5.68	6.51	7.51	7.84	7.08	7.93	6.99
Crude fibre	2.39	2.88	3.01	3.70	3.21	3.70	3.18	3.44
Starch	39.50	38.43	39.13	37.78	38.82	37.78	40.38	39.48

^1,^ C(1), control group; ^2^, D(2–5), experimental groups. Feed was commercial. Data is only analytical

### Growth performance

Body weight (BW) was measured on 5 dates of production: at stocking (day 1), and on days 10, 22, 35, and 42 (slaughter). We also recorded daily feed intake (FI) for each replicate in 5 groups, for each of the four feeding phases. Recorded data were used for the calculation of body weight gain (BWG) and feed conversion ratio per kg of body weight gain (FCR).

### Sample collection and meat quality analysis

After the rearing was completed, randomly selected chickens were slaughtered. The birds were starved for 12 h, and 10 birds from each group were selected. In order to identify the chickens, jiffy bands with ID numbers were attached to the wings. The birds were stunned with an electric current (loss of consciousness), and slaughtered by decapitation at the atlanto-occipital joint (rapid exsanguination). The carcasses were scalded in warm water at 60–65 °C, which allowed for the removal of all feathers. Feet were cut off at the ankle joints. Carcasses were gutted, and edible offal (heart, stomach, liver) were kept for further analyses.

The pH value of muscles was measured 45 min post-mortem (pH_45min_) using a pH-meter with a knife electrode (Elmetron, Zabrze, Poland) inserted in the greater pectoral muscle (*pectoralis major muscle*) at a depth of 2 cm. Carcasses were chilled and placed in a cold room (Hendi, Poznań, Poland) for 24 h at 4 °C.

After 24 h the pH of muscles (pH_24hours_) was measured again and the carcasses and edible offal were weighed (Radwag, Radom, Poland). Carcasses were dissected by separating the neck with skin, wings with skin, skin with subcutaneous fat, abdominal fat, breast muscles, leg muscles (thighs and drumsticks; without bones) and carcass remains (body and leg bones). All separated elements were weighed. Breast and leg muscles were used for further analyses. The colour of the right breast and leg muscles was analysed on the outer side of the muscles using a colorimeter CR400 (Konica Minolta, Tokyo, Japan) and the CIE (International Commission on Illumination) system (Martinez et al. 2020). For calibration the white calibration plate no. 21033065 and the D_65_Y_86_._1_x_0.3188_y_0.3362_ scale were used. Lightness (L*), redness (a*), and yellowness (b*) were analysed. After colour assessment, the right breast muscles were weighed and placed in plastic bags with a zip closure to analyse drip loss (loss of water in %) with minor modifications (Xing et al. 2020). The left breast and leg muscles from each group were disintegrated in a mincer (Hendi, Poznań, Poland). The water-holding capacity of minced muscles was analysed according to a procedure described by Biesek et al. (2020), and the initial weight of each sample was 0.300 g ± 0.005 g. The final measurement indicated the loss of water in percent. Additionally, the chemical composition of minced meat samples (90 g) was analysed using spectrometry (FoodScan, FOSS, Hilleroed, Denmark) and near infrared transmission (NIR) (Mendonca et al. 2020). The listed characteristics were tested for each of the selected birds.

### Statistical analysis

Obtained data were analysed with statistical software (Statistica Statsoft 2017). Means for each analysed trait were calculated for each treatment with standard deviation (± SD). The standard error of the mean (SEM) in total was calculated. The one-way ANOVA model was used to analyse variance. Differences in the values of the examined traits for each group were calculated. The significance of differences was verified using the post-hoc Tukey test. Analysis was performed using the one-way ANOVA model with consideration of the effects of subclasses. Differences were considered significant at *p* < 0.05*.* The experimental unit in the calculation of growth performance was replication of groups (10 × 10 birds). On the other hand, in the analyses of carcasses and meat quality, selected birds from each group were used in the amount allowing for statistical calculations. The number of birds used in the laboratory work is acceptable standard for poultry research.

## Results

### Growth performance

The losses of broiler chickens were controlled and recorded, but they did not exceed 1% in the flock. It was related to weak chicks at the beginning of rearing. The study revealed significantly higher body weight of broiler chickens in groups 3, 4 and 5 (LITTER: 3, 0.400 kg/m^2^ of halloysite and 0.400 kg/m^2^ of zeolite; 4, 0.800 kg/m^2^ of zeolite; 5, 0.200 kg/m^2^ of halloysite and 0.600 kg/m^2^ of zeolite; FEED: 3–5, proportion 25:75 of halloysite and zeolite, 0.5–2% in feed) compared to the control group (1) on day 22, and in groups 3–5 and 2 on days 35 and 42 (LITTER: 2, 0.800 kg/m^2^ of halloysite in litter; FEED: proportion 25:75 of halloysite and zeolite, 0.5–2% in feed) compared to group 1 (*p* < 0.05). The analysis of data on body weight gain showed significantly higher gains in groups 2–5 compared to group 1 on days 11–22, 23–35, and 36–42, and in the whole production period (days 1–42) (*p* < 0.05). Feed intake was significantly lower in group 1 compared to experimental groups, and for the whole production period it was highest in group 5 (*p* < 0.05). Feed conversion ratio (FCR) was significantly higher in group 5 compared to other groups on days 11–22, and in group 1 on days 23–35 (*p* < 0.05). No significant differences were found for other analysed growth performance parameters presented in Table 3 (*p* > 0.05).

**Table 3:** Growth performance of broiler chickens during the 42 days of rearing

Parameter	Group^1^					SEM	Significance
*N* = 100	1	2	3	4	5		
BW (g)							
1-day old chicks	43.83	44.10	42.80	43.92	42.89	0.20	NS
	± 1.24	± 1.60	± 1.60	± 1.31	± 0.98		
day 10	379.44	387.56	387.56	381.85	381.85	2.07	NS
	± 6.36	± 11.16	± 25.56	± 16.24	± 6.46		
day 22	1213.84^b^	1285.40^ab^	1294.27^a^	1289.96^a^	1289.18^a^	8.14	< 0.05
	± 36.70	± 31.91	± 70.21	± 69.21	± 28.13		
day 35	2095.00^b^	2423.92^a^	2472.66^a^	2476.85^a^	2471.70^a^	27.60	< 0.05
	± 128.74	± 69.54	± 136.96	± 190.18	± 101.11		
day 42	2348.79^b^	2867.59^a^	2970.348^a^	2993.83^a^	2996.03^a^	40.52	< 0.05
	± 161.94	± 86.78	± 185.20	± 130.14	± 144.43		
BWG (g)							
days 1–10	335.61	343.46	339.05	338.30	338.96	2.01	NS
	± 6.54	± 10.21	± 25.12	± 15.70	± 6.15		
days 11–22	834.39^b^	897.84^a^	912.42^a^	907.74^a^	907.33^a^	6.92	< 0.05
	± 34.36	± 26.64	± 51.40	± 56.09	± 24.95		
days 23–35	882.06^b^	1138.39^a^	1178.39^a^	1186.90^a^	1182.52^a^	20.98	< 0.05
	± 100.49	± 50.06	± 90.81	± 126.80	± 81.88		
days 36–42	252.89^b^	443.67^a^	497.68^a^	516.97^a^	524.32^a^	18.10	< 0.05
	± 102.64	± 64.75	± 64.51	± 78.72	± 85.31		
days 1–42	2304.96^b^	2823.49^a^	2927.55^a^	2949.91^a^	2953.14^a^	40.52	< 0.05
	± 162.03	± 86.10	± 184.42	± 130.01	± 145.87		
FI (g; per bird)							
days 1–10	343.35	353.76	370.51	361.43	335.32	4.09	NS
	± 17.62	± 28.23	± 35.40	± 26.03	± 25.43		
days 11–22	935.04^c^	1006.87^b^	1013.70^b^	1010.94^b^	1110.71^a^	10.08	< 0.05
	± 32.64	± 37.82	± 36.13	± 62.53	± 50.70		
days 23–35	1605.53^c^	1759.67^b^	1813.40^ab^	1864.13^ab^	1892.82^a^	18.95	< 0.05
	± 111.60	± 71.44	± 91.90	± 80.23	± 89.78		
days 36–42	508.33^b^	810.73^a^	800.16^a^	873.67^a^	860.60^a^	20.89	< 0.05
	± 68.15	± 66.06	± 40.94	± 46.24	± 77.47		
days 1–42	3541.02^c^	3938.02^b^	4170.21^ab^	4210.69^ab^	4312.98^a^	50.30	< 0.05
	± 213.23	± 138.35	± 301.86	± 240.00	± 230.99		
FCR (kg/kg)							
days 1–10	1.03	1.03	1.10	1.07	0.99	0.01	NS
	± 0.05	± 0.10	± 0.16	± 0.07	± 0.07		
days 11–22	1.12^b^	1.12^b^	1.11^b^	1.11^b^	1.22^a^	0.01	< 0.05
	± 0.04	± 0.04	± 0.04	± 0.08	± 0.06		
days 23–35	1.84^a^	1.55^b^	1.55^b^	1.58^b^	1.60^b^	0.03	< 0.05
	± 0.23	± 0.07	± 0.12	± 0.14	± 0.10		
days 36–42	2.36	1.85	1.62	1.72	1.67	0.08	NS
	± 1.16	± 0.23	± 0.15	± 0.29	± 0.24		
days 1–42	1.54	1.39	1.42	1.43	1.46	0.02	NS
	± 0.17	± 0.03	± 0.12	± 0.09	± 0.07		

a,b…, means in the same line with no common superscript differ between groups (*p* < 0.05); NS, no significance; ± SD, standard deviation; SEM, standard error of the mean for all data; ^1^, 1, no addition of aluminosilicates to feed or litter; 2, 0.800 kg/m^2^ of halloysite in litter; 3, 0.400 kg/m^2^ of halloysite and 0.400 kg/m^2^ of zeolite in litter; 4, 0.800 kg/m^2^ of zeolite in litter; 5, 0.200 kg/m^2^ of halloysite and 0.600 kg/m^2^ of zeolite in litter; groups 2–5, addition of halloysite and zeolite to feed (proportion 25:75; starter, 0.5%; grower 1, 1%; grower 2, 1.5%; finisher, 2%); ^2^, BW, body weight, g; BWG, body weight gain, g; FI, feed intake, g; FCR, feed conversion ratio, kg/kg

### Carcass traits

Table 4 presents data on carcass traits. Body weight and carcass weight in broiler chickens selected for slaughter were significantly higher in groups 2–5 than in group 1 (*p* < 0.05). Dressing percentage was significantly lower in group 4 compared to groups 1 and 5 (*p* < 0.05). The weight of neck with skin, wings, heart, liver and carcass remains (body and leg bones) were significantly higher in the experimental groups (2–5) compared to the control group (1) (*p* < 0.05). No significant differences were found for other analysed traits (*p* > 0.05).

**Table 4:** Traits of broiler chicken meat

Parameter *n* = *10*	Group^1^					SEM	Significance
	1	2	3	4	5		
Pre-slaughter body weight (g)	2211.50^b^ ± 198.02	3105.30^a^ ± 132.63	3109.00^a^ ± 235.19	3249.10^a^ ± 131.10	3092.90^a^ ± 119.84	58.34	< 0.05
Weight of carcass (g)	1734.27^b^ ± 161.56	2390.38^a^ ± 99.70	2346.22^a^ ± 204.15	2416.75^a^ ± 110.39	2354.88^a^ ± 95.60	41.55	< 0.05
Dressing percentage (%)	78.41^a^ ± 1.75	77.00^ab^ ± 1.49	75.47^ab^ ± 3.08	74.38^b^ ± 1.66	76.18^a^ ± 2.89	0.36	< 0.05
Weight and proportion in carcass							
Neck with skin (g)	90.18^b^ ± 16.23	120.28^a^ ± 11.18	124.34^a^ ± 16.20	120.86^a^ ± 12.35	126.86^a^ ± 13.25	2.69	< 0.05
Neck with skin (%)	5.22 ± 0.92	5.04 ± 0.52	5.30 ± 0.52	5.01 ± 0.52	5.40 ± 0.62	0.09	NS
Wings (g)	151.66^b^ ± 12.38	213.41^a^ ± 18.48	204.54^a^ ± 29.56	214.72^a^ ± 13.67	212.72^a^ ± 16.34	4.31	< 0.05
Wings (%)	8.78 ± 0.79	8.92 ± 0.64	8.70 ± 0.81	8.89 ± 0.55	9.03 ± 0.53	0.09	NS
Heart (g)	8.03^b^ ± 0.92	12.73^a^ ± 1.51	12.33^a^ ± 1.87	13.62^a^ ± 2.53	11.69^a^ ± 1.17	0.36	< 0.05
Stomach (g)	29.19 ± 6.11	35.51 ± 5.21	36.76 ± 10.20	35.35 ± 4.70	38.51 ± 9.56	1.11	NS
Liver (g)	39.67^b^ ± 7.30	57.66^a^ ± 6.05	57.11^a^ ± 10.12	62.04^a^ ± 10.32	60.85^a^ ± 10.24	1.68	< 0.05
Carcass remains (g)	457.11^b^ ± 68.97	603.48^a^ ± 73.31	587.96^a^ ± 68.37	660.02^a^ ± 80.61	586.65^a^ ± 51.68	13.36	< 0.05

a,b…, means in the same line with no common superscript differ between groups (*p* < 0.05); NS, no significance; ± SD, standard deviation; SEM, standard error of the mean for all data; ^1^, 1, no addition of aluminosilicates to feed or litter; 2, 0.800 kg/m^2^ of halloysite in litter; 3, 0.400 kg/m^2^ of halloysite and 0.400 kg/m^2^ of zeolite in litter; 4, 0.800 kg/m^2^ of zeolite in litter; 5, 0.200 kg/m^2^ of halloysite and 0.600 kg/m^2^ of zeolite in litter; groups 2–5, addition of halloysite and zeolite to feed (proportion 25:75; starter, 0.5%; grower 1, 1%; grower 2, 1.5%; finisher, 2%)

Table 5 presents data on the content of muscles and fat in broiler chicken carcass. The weight of breast muscles, leg muscles and total muscles (breast + leg) was significantly higher in the experimental groups compared to group 1. Moreover, the proportion of breast muscles in carcass was significantly higher in groups 2 and 3 compared to the control group (*p* < 0.05). The weight of skin with subcutaneous fat was significantly higher in groups 2 and 3 than in group 1, and its proportion in carcass and the proportion of total fat was significantly lower in group 4 than in group 1 (*p* < 0.05). The weight of total fat in the control group was significantly lower than in groups 2 and 5 (*p* < 0.05). In the Tables 2 and 3, the weight of individual elements, which may differ due to significant differences in the body weight of the birds, therefore the percentage share of the elements was shown for a more detailed analysis of the obtained results.

**Table 5:** Content of muscles and fat in broiler chicken carcass

Item	Group1					SEM	Significance
n = 10	1	2	3	4	5		
Weight and proportion in carcass							
Breast muscles (g)	512.89b	783.22a	771.18a	743.38a	725.59a	16.64	< 0.05
	± 67.91	± 34.54	± 58.76	± 64.97	± 84.72		
Breast muscles (%)	29.52b	32.78a	32.92a	30.73ab	30.76ab	0.32	< 0.05
	± 2.01	± 1.21	± 1.37	± 1.74	± 2.84		
Leg muscles (g)	374.08b	491.91a	489.65a	508.96a	521.55a	10.11	< 0.05
	± 50.09	± 30.19	± 73.53	± 43.75	± 39.07		
Leg muscles (%)	21.56	20.61	20.80	21.08	22.16	0.25	NS
	± 1.90	± 1.56	± 1.74	± 1.78	± 1.62		
Total muscles (g)	886.97b	1275.13a	1260.83a	1252.34a	1247.14a	25.06	< 0.05
	± 105.87	± 47.13	± 121.99	± 94.07	± 101.87		
Total muscles (%)	51.08	53.40	53.72	51.81	52.92	0.37	NS
	± 2.63	± 2.23	± 1.50	± 2.77	± 3.18		
Skin with subcutaneous fat (g)	163.78b	199.92a	196.32a	191.27ab	205.85a	4.06	< 0.05
	± 20.31	± 19.52	± 27.14	± 17.86	± 26.34		
Skin with subcutaneous fat (%)	9.43a	8.36ab	8.41ab	7.92b	8.73ab	0.14	< 0.05
	± 0.60	± 0.67	± 1.29	± 0.71	± 0.94		
Abdominal fat (g)	20.58	23.56	22.22	25.30	22.92	1.25	NS
	± 6.30	± 11.53	± 6.33	± 7.72	± 11.70		
Abdominal fat (%)	1.17	0.98	0.95	1.04	0.98	0.05	NS
	± 0.31	± 0.46	± 0.26	± 0.29	± 0.50		
Total fat (g)	184.36b	223.48a	218.54ab	216.57ab	228.77a	4.07	< 0.05
	± 25.10	± 22.80	± 24.26	± 23.72	± 29.24		
Total fat (%)	10.60a	9.34ab	9.35ab	8.96b	9.71ab	0.15	< 0.05
	± 0.73	± 0.72	± 1.14	± 0.89	± 1.11		

a,b…, means in the same line with no common superscript differ between groups (*p* < 0.05); NS, no significance; ± SD, standard deviation; SEM, standard error of the mean for all data; ^1^, 1, no addition of aluminosilicates to feed or litter; 2, 0.800 kg/m^2^ of halloysite in litter; 3, 0.400 kg/m^2^ of halloysite and 0.400 kg/m^2^ of zeolite in litter; 4, 0.800 kg/m^2^ of zeolite in litter; 5, 0.200 kg/m^2^ of halloysite and 0.600 kg/m^2^ of zeolite in litter; groups 2–5, addition of halloysite and zeolite to feed (proportion 25:75; starter, 0.5%; grower 1, 1%; grower 2, 1.5%; finisher, 2%)

### Meat quality

The value of pH_45min_ was significantly lower in group 3, where zeolite and halloysite were used in litter in a 1:1 proportion (400 g per m^2^) and in feed (25:75 ratio, 0.5–2%) compared to groups 1, 2 and 5 (*p* < 0.05). Yellowness (b*) of breast muscles was significantly lower in group 3 than in group 2 (*p* < 0.05). In the control group (1), water loss from breast muscles was significantly lower compared to experimental groups (*p* < 0.05). The content of protein in breast muscles differed significantly between groups, and in group 5 it was much lower compared to group 1. The content of collagen and salt in group 4 and the content of intramuscular fat and water in group 5 were significantly higher than in groups 1, 2 and 3 (*p* < 0.05) (Table 6).

**Table 6:** Physicochemical parameters of breast and leg muscles from broiler chicken

Parameter1	Group1					SEM	Significance
*n* = 10	1	2	3	4	5		
Breast muscles							
pH15	6.63a	6.68a	6.34b	6.47ab	6.62a	0.03	< 0.05
	± 0.22	± 0.19	± 0.17	± 0.17	± 0.20		
pH24	6.11	6.01	6.00	6.12	6.06	0.03	NS
	± 0.11	± 0.21	± 0.16	± 0.19	± 0.29		
Colour2							
L*	52.39	53.92	54.85	54.15	56.59	0.81	NS
	± 4.75	± 6.92	± 4.19	± 5.43	± 7.08		
a*	2.21	3.07	2.53	3.10	2.57	0.20	NS
	± 0.93	± 1.30	± 1.57	± 1.84	± 1.38		
b*	5.90ab	6.43a	3.94b	4.37ab	4.79ab	0.25	< 0.05
	± 1.51	± 1.84	± 1.77	± 1.49	± 1.27		
Water-holding capacity (%)	20.71b	29.82a	35.44a	30.21a	33.92a	0.91	< 0.05
	± 5.36	± 2.23	± 2.18	± 4.45	± 4.70		
Drip loss (%)	0.83	0.88	1.51	1.49	1.17	0.09	NS
	± 0.37	± 0.36	± 0.88	± 0.78	± 0.42		
Protein (%)	23.37a	22.67b	22.67b	21.65c	20.92d	0.12	< 0.05
	± 0.10	± 0.08	± 0.09	± 0.04	± 0.04		
Collagen (%)	0.71c	0.84bc	0.81bc	1.00a	0.97ab	0.02	< 0.05
	± 0.18	± 0.10	± 0.12	± 0.06	± 0.07		
Salt (%)	0.20c	0.18c	0.27b	0.38a	0.26b	0.01	< 0.05
	± 0.05	± 0.03	± 0.06	± 0.05	± 0.03		
Intramuscular fat (%)	2.53d	3.13b	2.83c	2.55d	3.40a	0.05	< 0.05
	± 0.05	± 0.05	± 0.03	± 0.02	± 0.01		
Water (%)	73.78e	73.90d	74.38c	75.45a	75.07b	0.09	< 0.05
	± 0.08	± 0.07	± 0.06	± 0.03	± 0.07		
Leg muscles							
Colour							
L*	49.06	50.39	51.80	47.74	51.04	0.61	NS
	± 3.15	± 4.02	± 3.39	± 4.05	± 6.08		
a*	2.52b	3.62ab	4.02ab	4.96a	3.28ab	0.21	< 0.05
	± 1.04	± 1.01	± 1.09	± 1.36	± 1.87		
b*	5.01	4.48	3.89	3.50	3.55	0.24	NS
	± 1.86	± 1.39	± 1.78	± 1.10	± 1.26		
Water-holding capacity (%)	38.61a	33.48ab	28.52b	37.41ab	36.69a	0.95	< 0.05
	± 6.03	± 1.27	± 9.09	± 2.61	± 6.62		
Protein (%)	19.08bc	19.46a	18.98bcd	18.93d	19.07bc	0.03	< 0.05
	± 0.05	± 0.06	± 0.13	± 0.02	± 0.06		
Collagen (%)	1.18	1.05	1.31	1.28	1.07	0.03	NS
	± 0.07	± 0.41	± 0.08	± 0.06	± 0.04		
Salt (%)	0.33	0.36	0.33	0.37	0.36	0.01	NS
	± 0.05	± 0.08	± 0.07	± 0.03	± 0.07		
Intramuscular fat (%)	8.49a	7.11c	7.99b	6.97c	5.93d	0.13	< 0.05
	± 0.44	± 0.04	± 0.07	± 0.04	± 0.02		
Water (%)	71.38e	72.56c	72.15d	73.14b	74.17a	0.13	< 0.05
	± 0.05	± 0.11	± 0.08	± 0.09	± 0.11		

a,b…, means in the same line with no common superscript differ between groups (*p* < 0.05); NS, no significance; ± SD, standard deviation; SEM, standard error of the mean for all data; ^1^, 1, no addition of aluminosilicates to feed or litter; 2, 0.800 kg/m^2^ of halloysite in litter; 3, 0.400 kg/m^2^ of halloysite and 0.400 kg/m^2^ of zeolite in litter; 4, 0.800 kg/m^2^ of zeolite in litter; 5, 0.200 kg/m^2^ of halloysite and 0.600 kg/m^2^ of zeolite in litter; groups 2–5, addition of halloysite and zeolite to feed (proportion 25:75; starter, 0.5%; grower 1, 1%; grower 2, 1.5%; finisher, 2%); ^2^, L*, lightness; a*, redness; b*, yellowness

The analysis of data on the physicochemical characteristics of leg muscles (Table 6) in group 4 revealed a significantly higher redness (a*) than in group 1, while in group 3 the loss of water (water-holding capacity) was significantly lower than in groups 1 and 5 (*p* < 0.05). In the group of chicken where 800 g of halloysite per m^2^ (2) was used, the content of protein in leg muscles was significantly higher compared to other groups of broiler chickens. The content of intramuscular fat in leg muscles from the control group was significantly higher compared to other groups, and the content of water was higher in group 5 (*p* < 0.05). No significant differences in other parameters were found between groups (*p* > 0.05).

## Discussion

A diet supplemented with natural zeolites (clinoptilolite) at the levels of 15 and 25 g/kg of feed had no significant effect on feed intake, body weight gain or feed conversion ratio in broiler chickens, while in the group where 25 g of zeolite per kg of feed was used feed intake was lower compared to the control group (Oguz and Kurtoglu 2000). No differences in body weight gain or in the feed conversion ratio were found in broiler chickens fed a diet with the addition of hydrated aluminosilicates at the level of 5 g/kg of feed in a study by Prvulovic et al. (2008). In our study, supplemental aluminosilicates also had no effect on the feed conversion ratio, but feed intake was significantly higher in experimental groups compared to the control group, and body weight and its gains were greater in the groups where aluminosilicates were used. Karamanlis et al. (2008) also reported a positive effect of zeolites in feed and litter on the growth performance of chickens, and even on the quality of their litter. The differences in the obtained results could be caused by the different proportions of specific types of aluminosilicates and their form, as well as the method of application (in feed and/or litter), and even the type of litter used in the production of broiler chickens. Higher feed intake is associated with higher body weight, and the positive effect of aluminosilicates on performance parameters may depend on the reduced levels of toxic substances present in the farm building or even in the feed (Al-Nasser et al. 2011). Another study revealed that halloysite reduces the contamination of feed (Kulok et al. 2005).

A study investigating the effect of 1–1.5% of sodium bentonite or calcium bentonite found no significant effect on broiler chicken carcass traits (Khanedar et al. 2012). Similarly, nanostructured bentonite did not influence chicken carcass traits, apart from an increased proportion of abdominal fat, but it reduced the negative impact of aflatoxins in feed on growth performance parameters (Keyhani-yazdi et al*.* 2019). In our study the weight of fat in carcass was higher in groups that received aluminosilicates in feed and 800 g/m^2^ halloysite (2), and the combination of two aluminosilicates at the level of 200:600 g/m^2^ in litter (5). In group 5, the content of intramuscular fat was higher in breast muscles, but in leg muscles from each experimental group its content was lower compared to the control group. Damiri et al. (2012) reported a positive effect of aluminosilicates on carcass traits in chickens. We found significantly higher weight of breast and leg muscles, but no significant changes in dressing percentage, which indicates that such results were obtained because of body weight gain. Interestingly, the weight of liver was higher in chickens where aluminosilicates were used. The lower weight of liver in birds receiving higher doses of sodium bentonite was associated with binding toxins (Damiri et al. 2012). It can be assumed that in our study the level and type of supplemental aluminosilicates influenced the quality of feed. Other factors to consider include the chemical composition of feed, as well as the metabolism of lipids and their accumulation in the liver (Karimirad et al. 2020).

A comparison of our findings on the pH of breast muscles with data reported by Qiao et al. (2001) leads to the conclusion that the pH of muscles was optimal in all groups of chickens, and the obtained meat should be regarded as normal, but the value of lightness (L*) indicates that meat colour was between light and normal. According to Hertanto et al (2018), tissue acidification values between 5.81—6.30 are for normal meat. However, after 24 h, the meat should have a pH close to 6.00. Consistently with our study, Hashemi et al. (2014) demonstrated that the addition of zeolite was associated with a greater loss of water from breast muscles and leg muscles. Our research revealed a positive effect of zeolite and halloysite in litter (400 g/kg for both) and in feed (0.5–2%) on water-holding capacity of leg muscles. Meat quality parameters (including WHC) and the chemical composition of muscles may depend on protein oxidation (Zhang et al. 2013) and nutrients used for the preparation of feed (Olfati et al. 2020).

The effects of aluminosilicates were also investigated in laying hens and other animal species. Improved quality of eggs (Skiba et al. 2009), and positive effect on fatty acid composition in pigs were found (Korniewicz et al. 2006), but also the positive effect of zeolite on the quality of meat from broiler chickens and halloysite in feed and litter on the intestinal morphology (Banaszak et al. 2020).

Summing up, the results obtained in the experimental groups where we used halloysite and zeolite in a 25:75 ratio in feed at the level of 0.5–2% in different feeding phases (starter, grower 1 and 2, finisher) and in litter (chopped rye straw) at different levels and proportions (2, 0.800 kg/m^2^ of halloysite in litter; 3, 0.400 kg/m^2^ of halloysite and 0.400 kg/m^2^ of zeolite in litter; 4, 0.800 kg/m^2^ of zeolite in litter; 5, 0.200 kg/m^2^ of halloysite and 0.600 kg/m^2^ of zeolite in litter) indicated that these aluminosilicates can improve body weight gain without increasing feed conversion ratio. We also found no negative effect of aluminosilicates on most of the analysed physicochemical parameters of meat or the quality of breast and leg muscles, except the water-holding capacity of breast muscles. Importantly, the use of the proposed aluminosilicates was associated with higher proportion of muscles in chicken carcass. In practice, aluminosilicates in poultry production could increase the live body weight of birds, which is one of the most important aspects from the producers’ point of view.

## Data Availability

The datasets generated and analyzed during the current study are not publicly available due to all the results in the form of means and statistics are presented in this paper, but are available from the corresponding author on reasonable request.
